# Supplementary material for: Membrane Charge Effects on Solute Transport in Nanofiltration: Experiments and Molecular Dynamics Simulations
Source: Membranes (Basel). 2025 Jun 18;15(6):184. doi: 10.3390/membranes15060184 (PMC12194865; doi:10.3390/membranes15060184)
Supplement: Supplementary file 1 [file membranes-15-00184-s001.zip › membranes-3661347-supplementary.pdf]

Supplementary Material

Membrane Charge Effects on Solute Transport in Nanofiltration Membranes: Experiments and Molecular Dynamics Simulations

Suwei Liu, Zi Hao Foo, John H. Lienhard, Sinan Keten,
Richard M. Lueptow

Table S1: Membrane characterization for all membrane models

Name	Net Charge	Charge Composition	Average Density [g cm^{-3}]	Mean Pore Diameter [nm]
pH 2	+14 *e*	14 NH_2^+	0.79	5.9
pH 7	−14 *e*	14 NH_2^+, 28 COO^-	0.76	5.8
pH 10	−30 *e*	30 COO^-	0.83	5.0
flip	−14 *e*	14 NH_2^+, 28 COO^-	0.76	5.7
bilayer	−14 *e*	14 NH_2^+, 28 COO^-	0.78	5.9
feed	−14 *e*	14 COO^-	0.85	5.6
center	−14 *e*	14 COO^-	0.84	5.0
permeate	−14 *e*	14 COO^-	0.79	5.6

Table S2: Ion rejection measurements with the NF 270 membrane based on binary cation solutions, at solution pH of 2, 4, 7, and 10.

Feed Molarity [M]	Feed pH [–]	Feed Pressure [bar]	Temperature [°C]	Water Flux [LMH]	Species Rejection [–]		
					Na^+	Ca^{2+}	Cl^-
0.13 M NaCl							
0.130	2	10	20.0	86.45	0.481	–	0.455
0.130	2	15	20.0	140.63	0.498	–	0.462
0.130	4	10	20.0	145.83	0.151	–	0.177
0.130	4	15	20.0	227.08	0.178	–	0.184
0.130	7	10	20.0	130.21	0.207	–	0.209
0.130	7	15	20.0	198.96	0.209	–	0.194
0.130	10	10	20.0	102.08	0.296	–	0.305
0.130	10	15	20.0	169.79	0.299	–	0.326
0.13 M $CaCl_2$							
0.130	2	15	20.0	60.41	–	0.926	0.817
0.130	2	22	20.0	110.47	–	0.915	0.830
0.130	4	15	20.0	73.96	–	0.541	0.600
0.130	4	22	20.0	112.50	–	0.615	0.676
0.130	7	15	20.0	84.38	–	0.479	0.534
0.130	7	22	20.0	132.39	–	0.483	0.550
0.130	10	15	20.0	94.78	–	0.455	0.511
0.130	10	22	20.0	139.58	–	0.422	0.481

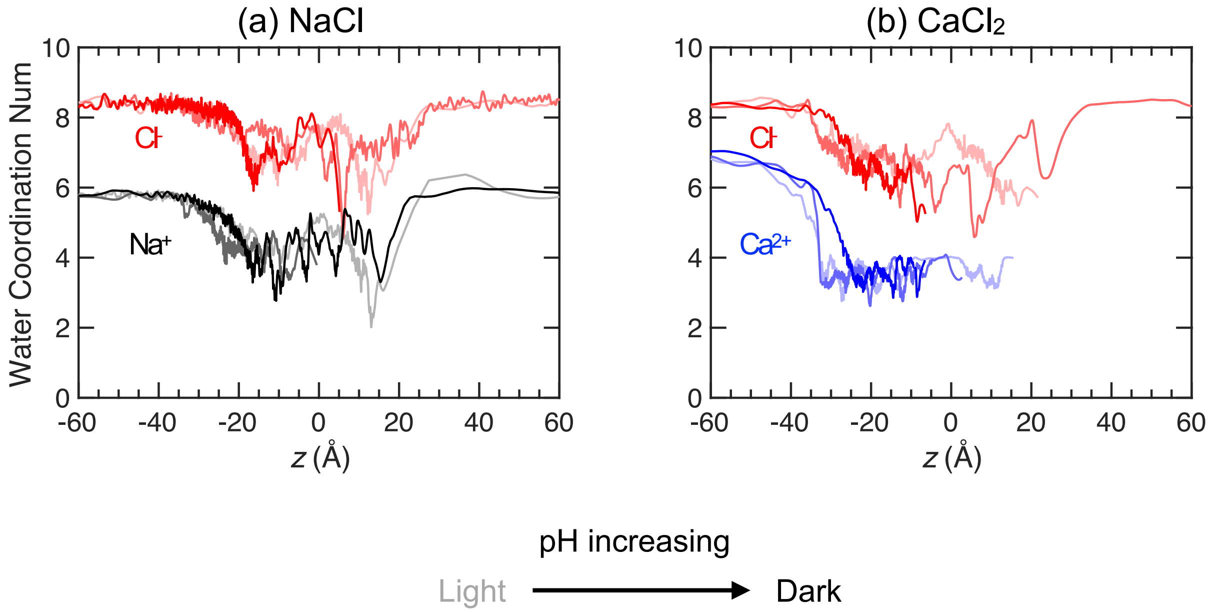

Figure S1: Water coordination number in the *z*-direction of membrane thickness across pH = 2, 7, and 10 for (a) NaCl and (b) $CaCl_2$ feeds, where different shades of black curves indicate Na^+ ions, different shades of blue represent Ca^{2+}, and different shades of red represent Cl^- at various pH levels; the darker the color, the larger the pH. (Color online.)
